# An Essay on the Preparations of Iodine

**Published:** 1844-11

**Authors:** John O’Ferrall

**Affiliations:** Ohio


ART. II.—*An Essay on the Preparations of Iodine.* By JOHN O'FERRALL, Jr., M.D., of Ohio*.

The deleterious influences which were so frequently observed to arise from the free use of Iodine, led Dr. Coindet to institute a series of experiments by which he might if possible ascertain the therapeutical properties of the drug without its attendant danger. The result of his experiments was the recommendation of the ioduretted solution of potassa for in-

---

* This Essay was submitted to the Officers of the Medical Institute of Louisville, for the Degree of M.D. in February, 1844.

ternal, and the iodide of potassium for external use. His suggestion however was not received with general approbation until recently; the use made of the latter compound among the profession at large has been as a solvent for free iodine, or when applied externally, the internal use of free iodine has generally been conjoined with it, and *vice versa.*

One set of practitioners administer the iodine by drachms, while the experience of their contemporaries will not warrant them in exceeding the same number of grains per dose. Idiosyncracy added to the varied conditions of the constitutions of individuals at different times, and numerous considerations of a chemical nature, may be the cause of so much diversity of opinion between practitioners and writers.

Though a powerful remedy, it acquires its sway by slow, but certain approaches, not like strychnine, or arsenic which cannot be pushed beyond very limited doses, without at once reminding the practitioner that he is dealing with edge tools. Its action on the animal economy, in a great degree, resembles that of mercury: they both promote the excretory functions, and it is in this way, most probably, that they increase the activity of the assimilating functions; it appears to be more general in its operation on the system than mercury; its action on the uterine system is decided; on the secretory functions of the liver, pancreas, kidneys, and skin it is also quite marked; it thus diminishes abdominal plethora, promoting the action of the eliminative, and through them, of the assimilative functions.

There seems to be two opinions in regard to the manner in which the agency of iodine, chemically combined, is exerted on the human system. Some are of the opinion that iodine, and its compounds all follow essentially the same law of action—that is to say, when iodine is indicated it is a matter of little importance which preparation of the drug is used, while on the other hand, we find it maintained that the compound in question, combines the properties of the substance united to the iodine with those of the iodine itself.

To draw a comparison between the effects of the iodide of potassium and iodine itself is a matter of very considerable difficulty. That they present much resemblance in their operation and effects, is generally admitted, as we may infer from the vague manner in which they are indiscriminately spoken of, the term iodine being applied as much to the one as to the other.

This salt as admitting of larger solution in water, and being less liable to irritate the mucous tissues, is very generally entitled to a preference over iodine in substance, syrup, or tincture, and there is scarcely a disease to which the drug, in a simple state, is applicable, that has not been cured by this compound. Still there is considerable difference in their mode of operation, and that not altogether referable to their relative powers of producing irritation. The iodide appears to be much slower in its action on the system than iodine, and in the hands of some it has failed in several diseases in which the employment of iodine has been attended with eminent success: the want of success was probably owing to the mode in which it was used.

The compound has also proved to be preferable to iodine in some diseases, and the success attending its use greater, as in dropsy and diseases of the kidneys attended with albuminous urine, &c.: other points of difference will be shown in subsequent pages. The bad effects which occasionally arise from the free use of this substance may in a great measure be refered to its adulterations, and the chemical changes which take place in it in the alimentary canal; even most articles of ordinary nutriment may often be suspected of modifying or changing its action. Are we not taught by experience that the same substances which in moderation exert a bland and wholesome influence, may when pushed beyond bounds, produce injury and destroy life?

These and other causes which will be spoken of in another part of this essay may explain the extraordinary discrepancy of opinion which has existed among medical men in reference to the true character of this compound.

In regard to this salt it should be observed that a preternatural sensibility or irritability of the system, a tendency to irregular determination, the presence of internal local congestion, prominent gastric or intestinal derangement, general plethora, febrile symptoms, hemorrhage, diarrhœa, and an inflamed and sensible state of tumors, are decidedly counter-indications to its employment, whether externally or internally. In its action on the animal economy it generally directs its power on tumors and diseased structures, not interfering with the healthy parts of the body.

*Some of the effects which are seen to arise from the use of this compound.*

To a female who swallowed an ounce and a half of the ioduretted solution of the iodide of potassium, the consequences were general uneasiness, nausea, acute pain, and burning heat in the stomach, followed by vomiting, headache and vertigo: recovery took place after the lapse of several days. This case is certainly not sufficient to establish any well defined inference, because the effects of the combination of the iodine with the iodide of potassium cannot be correctly distinguished. Still it has its value, if it is only to show that the combination acts energetically.

Two patients, who, on the same day, took a drachm of the iodide in divided doses, were attacked with nausea, vomiting, colic pains, slight diarrhœa, frequency of the pulse, and general exhaustion—both were affected precisely in the same way.

The accounts which have just been given of its effects would, in the absence of contradictory evidence bring the iodine under Mead's definition of poisons. Devergie considered it as a poison, and stated that the fatal dose was a little more than that of iodine: he estimates the last at fifteen or twenty grains. Doubtless his idea of its virulence was in some degree exaggerated; be this as it may, it serves to increase the perplexity, and render the transition to the opposite side more startling.

Dr. Elliotson, whose enlightened efforts have tended greatly to improve our acquaintance with the proper limits of many remedies, finding that two or three grains of this substance taken daily, as was generally ordered, were ineffectual in certain chronic affections, began in a case of scirrhus uteri to administer it in drachm doses several times per day: the result was relief from pain, &c., unaccompanied by sickness or any other unpleasant feelings.

In several cases of scrofula he gave it in six drachm doses per day, for weeks, without any bad effects. Magendie and others have also used it in as large doses as the above without any deleterious effects.

Dr. Buchanan tells us that the half of an ounce may be given at a dose without producing pain of the stomach, purging, or any hurtful effects.

An ounce or more has been given in Paris by some of the practitioners of that city without bad results. Still we may readily suppose whilst receiving implicitly these statements as true, that they evince the tolerating power of some persons more than the propriety or even safety of administering the iodide in such large doses as a general rule. When we compare these last statements with the accounts which immediately preceded them, it must be owned that they draw largely on our credulity.

The article which was administered in such large doses may have been impure.

In numerous instances where physicians were in the habit of using this salt with the utmost freedom, asserting that no bad consequences had occurred from it, on proper inquiry being made into the composition of the article used by them, it was found to be very impure.

These chemical facts might have been sufficient to explain the mystery, if we had not been assured by respectable authority that means had been taken to purify the drug before using it.

That consequences so different, and sometimes opposite, should arise from the use of the same article is a point on which the mind is not easily satisfied. From ten to twenty grains dissolved in water or some other proper vehicle, given two or three times a day, is a quantity which if continued for a sufficient time, will prove to be equal to the exigencies of any case in which it is applicable. Dyspeptic symptoms, restlessness, headache, vertigo, and slight salivation, are the most common bad consequences which arise from the long continued use of it. Sometimes it seems to produce constipation, at other times relaxation of the bowels.

But the curative effects proceed in the generality of cases independently of these or any other less important consequences, and persons who are under the full influence of it usually require no particular care except as relates to diet.

Of the much dreaded atrophic influence of this substance on the generative organs, but few cases are on record. Dr. Hill cites one in which both mammæ were absorbed in a patient affected with cancer of the breast; one or two cases of absorption of the testicle are also on record; it sometimes destroys the functions of those organs without causing their absorption. A peculiar kind of coryza, characterized by pain in the nose and forehead, and an increased flow of mucus from the nasal passages, occasionally arises from its use. It is said to augment the flow of blood to the lungs and trachea, causing increased secretion of mucus or hæmoptysis, and in some instances rouses to a state bordering on inflammation.

I am led to believe that such effects very seldom, if ever, occur from the cautious use of the iodide of potassium; perhaps from excessive doses, and peculiarity of constitution such bad results may occasionally arise. Its deleterious action on the nervous system has been already partially referred to: the most common are restlessness, headache, vertigo, muscular tremors, and want of sleep, which generally occur in weak and debilitated persons, and such as have used it for a long time, and in large doses. On the surface of the body this substance produces much less remarkable effects than iodine.

In a number of patients on whom Lugol and others tried the influence of baths containing an ounce or more of this substance, nothing but temporary itching was produced; by others in the form of ointment it has been found sufficiently active to produce inflammation, ulceration, and the final eradication of tumors; a very considerable degree of smarting is no uncommon circumstance arising from the external use. Dr. Gardner states from personal experience or observation, that its absorption through the cuticle relieved tumors in distant situations, and that the supervention of gastric pain and copious bilious diarrhœa occasionally took place.

Small warty excrescences attended with extreme itching and pain occasionally arise.

From the experiments of Devergn on the lower animals, we are led to conclude that it is a substance which if placed beneath the skin will produce irritation, inflammation, and death, and it may be detected in the various secretory organs of the body of the animal after death. John Davis, M.D. thinks, as an external application for the relief of disease, the iodide of potassium is objectionable, in consequence of the potassium rendering the iodine more acrid, and giving it a tendency to inflame the skin, causing a very considerable degree of smarting when brought into contact with an ulcerated surface.

It frequently causes desquamation of the cuticle; this only becomes observable sometime after its use is discontinued, and the skin has been suffered to become dry. The desquamation of the cuticle more generally arises when the ioduretted solution of potassium is used, proving that the degree of energy with which it acts on the skin, is in proportion to the amount of iodine in solution. Dr. Peschier, of Geneva, observes that slight pain of the stomach accompanied by copious bilious discharges, generally presented itself in patients using this compound externally.

The experiments instituted by Lugol in reference to the respective action of the iodide of potassium and the ioduretted solutions of potassa in baths, are the most conclusive and unexceptionable of any which have been presented to my consideration. From the results obtained by him we are led to consider the iodide of potassium as a mere neutral ingredients; serving to dissolve the iodine and not otherwise participating in the therapeutical agency of the baths.

The first and second series of experiments made by him with free iodine in the baths leave no room to doubt, that when prepared without being previously dissolved in a solution of the iodide, they contain the iodine partially in suspension, and consequently do not present it to the skin in a state of sufficiently minute division. And the bad effects which followed from the use of the alcoholic tincture of iodine in the baths were more marked than the above. Thus, the fact is established, that it is actually necessary for the iodine to be dissolved in a solution of the iodide in order to secure its equal and complete division in the ordinary quantity of water for a bath, and by this combination its action becomes more equal, and above all it can be thus more accurately graduated. He chose seven patients for his experiments, and the results deducible from the effects produced on them are,

1st. The iodide of potassium has scarcely any action on the skin in the quantity not exceeding three ounces in a bath.

2d. Iodine should be regarded as the active principle.

3d. The proportion of iodine in general should not exceed two or three drachms.

4th. Iodine is not completely soluble in water in its simple state, consequently it is liable to excite great local action.

5th. Previously dissolved in alcohol it does not continue in a state of solution, when mixed with water in bath, thus increasing the liability to local action.

6th. The most certain and least objectionable method of preparing the baths, is the previous solution of the iodine in a solution of the iodide of potassium.

One fact in regard to the external use of the iodide is, that in proportion as the cure progresses its power of producing pain and irritation decreases.

Mr. Pereira states it as his belief, that but one house in London, some years since, sold the pure iodide of potassium. Out of twenty specimens examined by Dr. O'-Shaughenessy only two were free from adulteration. The carbonate of potassa is sometimes sold for the iodide. The chloride of potassa is sometimes added for imposition.

An easy method for detecting the carbonate, consists in adding to the suspected article, the tincture of iodine; if the carbonate be present the tincture will lose its color; also by immersing a crystal of the suspected salt in lime water, the presence of the carbonic acid will be indicated by the water becoming milky.

The test for the chloride of sodium consists in adding nitrate of silver to the suspected article; any carbonates, chlorides, and iodides are thrown down; digesting the precipitate in ammonia, you take up only the carbonate and chloride; adding nitric acid to the solution, the carbonate is converted into the nitrate, and the chloride is precipitated.

*In scrofula.*—The success attending the judicious employment of the iodide of potassium in the various grades of this disorder, is so remarkable, that a disease of the utmost prevalence in this and other countries; one which formerly proved to be very protracted, and difficult in its treatment, and one which was considered by many incurable, has come to be perfectly under the control of the practitioner. I might cite in illustration of its curative power, not only cases of the first and second grades of scrofula, but also those in which that disease existed in the most aggravated form. It has effected cures in extensive tubercles of years' standing, of strumous ophthalmia, coryza, extensive ulceration of the cellular system, also of induration and hypertrophy of the same, extensive disease and ulceration of the cutaneous system, white swelling of the knee, hips, shoulder, elbow, &c., attended with caries of the bone.

*In bronchocele.*—Dr. Coindet seems to have been the first person who used the iodide with success, since which time its curative power over this disease has been well established; still the proofs of this power, for reasons already assigned, are not so numerous as are those of the power of iodine. He was so well satisfied with the results obtained from applying it externally as to be fully warranted in recommending this as the only unexceptionable method of using iodine. Many do not agree with him in consequence of finding such a plan by itself not sufficiently active, and they combine the internal use of it with the local.

A great number of cases illustrative of its specific power over this disease might be quoted from authors, several of whom deemed it scarcely necessary to pay any attention to other remedies which were formerly recommended for the cure of this affection, and it can hardly be presumed, that where the judicious employment of the iodide fails, there can be any particular advantage derived from any of the remedies at present known.

It has not proved to be equally efficacious in all cases of scrofula; no plausible reason for this difference in its action has been assigned.

*In phthisis pulmonalis.*—The beneficial effect of the iodide of potassium in scrofula affecting the external parts, induced the hope that it might also prove useful in consumption.

In some constitutions it does not appear to produce any obvious effect either for the better or worse; but in the majority of cases, even in the second stage, its curative operation is quite obvious. The strongest testimony that I have met with to its utility in phthisis is given by Dr. Morton. He states, that having used it extensively, he is able to express an uneqivocal opinion respecting its power. In a large number of instances it has appeared, especially in the incipient stage, to arrest or suspend the tubercular secretion, and with it the hectic, marasmus, cough, dyspnœa, and other urgent symptoms. It also often improves the complexion, and restores the appetite, even when there is no hope of recovery, owing to the advanced stage of the disease.

It so obviously improves the nutritive functions in some cases, that patients have increased in flesh, strength, and spirits, by its use, and at the same time recovered to a considerable degree a natural florid complexion. The results of Dr. Bardsley's observations on the use of this drug in this disease are very different from the last.

He employed this medicine in fifteen well marked cases of incipient phthisis, with a strict attention to its effects; in five instances it appeared to arrest the further progress of the disease, but the apparent amendment was only temporary; the disease finally proving fatal.

Dr. Baron says, that he has reason to believe that tuberculous disease of the lungs in the incipient stage has been removed by the judicious use of this compound. Mr. Cooper relates three cases in which marked improvement was perceived in a few weeks after commencing the use of the iodide, and an effectual cure in a few months; the hereditary predisposition to this disease was well marked in all the cases reported by him. I have tried this salt in three well marked cases of incipient phthisis, and with decided benefit in two of them, the cough, dyspnœa, night-sweats, pain in side, &c., having been relieved in the course of a few weeks, and not having since returned. In the other case it seemed to hasten the suppuration of the tubercles, and consequently the death of the patient.

The use of the iodide was continued for some time after recovery in the cases to which I refer. In the second of the three in which this solution proved of advantage, there had been for some time previous to his applying to me a very considerable quantity of purulent matter expectorated. His recovery perhaps may have been owing to his strength of constitution, the predisposition to the disease in him not being very marked.

The other one in which its curative effects were more decided, had suffered several attacks of hæmoptysis, and was very weak, which protracted the cure. He is now following his occupation as a farmer, and enjoying fine health, without the least indication of the further progress of his much dreaded malady. In this case the predisposition was very strong. It has been six months since he was discharged as cured.

The tubercular disease of the lungs is only a secondary affection, the consequence of a pre-existing morbid state of the constitution, generally a hereditary one, sometimes acquired by various causes independent of a hereditary predisposition; and he who is acquainted with the morbid anatomy of it cannot for a moment indulge the hope of ever being able to cure confirmed consumption, if we except the small number in which the deposit is confined to a very limited portion of the lung. A hope may be entertained of diminishing the hereditary predisposition, by investigating the circumstances in the health of the patients which predispose their offsprings to the affection.

Childhood and youth seem to be the periods of life in which tuberculous complaints are most rife, and fortunately they are those in which much may be done to destroy the tendency to disease. Judging from the effect this substance has had on the few adults, on which it has been used for the cure of phthisis, we may conclude that judiciously emplyed in the young who have a predisposition to consumption, it may be found a remedy of great value in preventing the secretion of the morbid matter, more especially if used at that period in which the tuberculous cachexia exists. In many cases, no doubt, the result will be fatal in spite of all human aid; still there are many in which the progress of the disease may be suspended, and life prolonged for a considerable time; and in a few where the hereditary or acquired predisposition to the deposit of tuberculous matter is slight, it may effect a cure.

*Secondary and tertiary syphilis.*—Of all diseases constitutional syphilis is perhaps the one which most decidedly requires the prompt interference of art. The first symptoms which make their appearance after chancre should be promptly treated, for the serious nature of syphilis depends in a great degree on the time allowed it to undermine the system.

The iodide of potassium has been substituted by some physicians for mercury, in the treatment of syphilis in all its forms. It is also said that only two cases out of every ten of chancre are followed by secondary symptoms if left to nature. If such is the fact, it proves that eight out of every ten persons with chancre in common practice, are mercurialized without any necessity. This is a very great evil to the scrofulous, and consumptive, and as mania, erysipelas, palsy enteritis, &c., occasionally arise from its use, we should not employ it more frequently than is actually necessary.

Eleven cases of tertiary syphilis are reported by Dr. Bullock, in which this compound effected a perfect cure in a limited time. Dr. Wallace used it in one hundred and forty cases, and with what degree of success I have not been able to ascertain, but judging from the manner in which he speaks of it, it was of no ordinary kind. Professor Gross and others consider this substance as much a specific for this disease as quinine is in intermittent fevers.

Among the various causes which now and then produce looseness of the teeth, none is more common than inflammation of the alveolar processes and sockets. Sometimes this originates in a disease of the teeth themselves or of the gums, but in other instances the diseased action commences in the alveolar processes, and by spreading to the socket and gums it gives rise to great pain, swelling, and sponginess of the gums, and eventually involves the fangs of the teeth, and detaches therefrom the grasp of the sockets and they fall out. The progress of the disease is accompanied by extreme pain, and a purulent discharge oozes out from between the teeth. In several such cases the iodide has been used with entire satisfaction, effecting a cure in a very limited time.

In gonorrhœa the iodide of potassium has been used in quite a number of cases, most generally with success. It has proved more especially beneficial in the chronic variety, many cases of which might be quoted which were cured by it.

In chronic laryngitis, bronchitis, tonsilitis, &c., it is a remedy of the highest value; many cases of each might be cited in which its use was followed by success.

In amenorrhœa this salt has, in the hands of several physicians, proved a remedy of very considerable value, and been successful in most cases in which it was used.

Several young women to whom Dr. Bardsley gave the iodide did not menstruate until their sixteenth year—he thought that process was retarded in consequence of the use of this medicine. The opposite effect has been observed. Dr. Wallace gives one instance in which a female, who had not menstruated for several years, became robust, and menstruated after using this compound for a short time.

John Bell, M.D., says "as an emmenagogue I can certify to its powers." He has also found its administration for a short period, followed by such an amended condition of the constitution in married women, who had been before its use childless, that they soon conceived, and had healthy children. It has been successfully emplyed in chronic diseases of the uterus, attended with induration and enlargement.

In dysmenorrhœa its success has also been very flattering. In leuchorrhœa this compound has exerted a curative power of the most striking nature. Several cases which had proved to be unmanageable by the use of the remedies commonly resorted to, were cured by it in a few weeks.

Chronic enlargements of the liver, spleen, pancreas, prostate gland, &c., are very often materially relieved, if not cured, by the use of the iodide.

In rheumatism, more especially in the chronic stage of the disease, it is one of the most powerful remedies we possess, and is considered by many of our most respectable practitioners and authors as a specific.

Dr. Elliotson, who seems to have been the first who used it in this disease, administered it in doses of from five to eight grains, three times a day, with results highly flattering. I might, if it were deemed advisable, give the particulars of several very interesting cases, which have lately fallen under my care, in which this compound relieved effectually all the symptoms in a very short time. In these cases it was given in doses of from ten to twenty grains three times a day, according to the effects produced. In several cases in which the disease was merely local, the ointment was used in connexion with the internal administration of the remedy.

In dropsies, especially such as are attendant on, or caused by enlargement or chronic inflammation of the liver or spleen, it has been found to be of very material benefit. Cumming, Bain, Ricord, and others report cases of the different varieties of dropsy cured by it. Dr. John Bell speaks very highly of the iodide in a variety of dropsies, attended with disease of the kidneys, in which albuminous urine is secreted.

A case of anasarca which fell under my observation lately, in which squills, digitalis, purgatives, &c., had been used with very little or no apparent benefit, was effectually cured in two weeks by giving ten grains of the compound three times a day. The patient was salivated when we commenced the use of the substance, but the ptyalism was perfectly relieved in a few days.

In a case of chronic diarrhœa in which the attending physician had tried all the remedies commonly resorted to without the least benefit, the iodide in five grain doses, three times a day, effected a cure in a few weeks. This case was of long standing, following a very severe attack of fever, for which the patient had been salivated.

This compound, according to the results obtained by Dr. Bardsley, seems to have less influence over nervous diseases than iodine. His want of success in these affections may have been owing to the smallness of the doses which he used, for Dr. Manson and others found it to be eminently beneficial in all the cases in which they used it.

That it exerts a powerful influence on the nervous system I am well assured from observation. It has produced in some instances a feverish excitement, headache, wakefulness, giddiness, &c.

In several cases of dyspepsia of very long standing, for the cure of which all the remedies which had from experience been found to be most useful were ineffectually tried, the iodide was finally used, and with entire success.

The curative effects of a strong solution of this substance and iodine can only be appreciated by those who have received benefit from it, two or three applications being generally all that is needed to relieve the pain, &c., of inflammation. It has been used with success in inflammation of the absorbents, in chilblains, malignant ulcers of the tonsils, and tongue, whitlow, burns, scalds, &c.

Lastly, it promises the power of causing determination of diseased action to the skin. In cases of suppressed measles and scarlatina, it will frequently induce healthy reaction under the most desperate circumstances.

February, 1844.

